# Ohio State Medical Society

**Published:** 1875-06

**Authors:** 


### Miscellaneous.

### Meeting of the Ohio State Medical Society.

The annual meeting of the Ohio State Medical Society was held at Put-in-Bay, Tuesday, Wednesday and Thursday, June 15, 16 and 17, Dr. W. W. Jones, of Toledo, presiding.

We make the following extracts from his address:

GENTLEMEN:—The position with which you have honored me, brings with it cares and duties, and is entered upon with distrust in my ability to meet the expectation which the station involves. I embrace the trust with greater satisfaction, because it has been bestowed by the profession of a State which has always been foremost in earnest endeavors to contribute to the advancement of medical science.

We are engaged in a struggle to assist nature in overcoming the evils, which disobedience of the organic laws impose upon mankind. Our measure of success depends upon the knowledge we possess regarding her processes, and the resources with which we are supplied.

Unlike the professions of Law and Divinity, the former of which deals with the rights and wrongs of the individual, and the latter with his duties towards his fellow and his Creator, axioms which imply a degree of knowledge only attained at the age of mental accountability, ours ministers to the earliest manifestations of his organization, and is the Alpha and Omega to which mankind look with imploring eyes when sickness or death invade the habitation of the soul.

Medicine does not claim to be an exact science, for the reason that it involves facts and problems which lie beyond the field of observation. The long history of medicine is fruitful in attestation of its struggles with the unknown, showing that errors which have gained favor in one age have been overturned by the next.

While progress in medicine must be slow from the very nature of things, we are assured that the present age is witnessing a more substantial and rapid progress than those which have preceded it. The general use of the microscope, opthalmoscope, stethescope, laryngoscope, speculum, aspirator, and the many other valuable means almost unknown to a former generations, have wonderfully increased our power for observing and recording facts, and made the verification of those facts of easy demonstration the world over. Thanks to the "republic of letters," no national boundaries or tariffs impose barriers upon the free interchange of thought, opinion and discovery.

With these preliminary remarks, I propose to call your attention to some of the means within the control of this society for promoting progress in our art.

That such has been accomplished by this society during the twenty-nine years of its existence, is well known to the older members of the profession, who remember what was the state of medical and surgical practice before it organization. While we have been greatly aided by the National and other State organizations, and perhaps, more than all, by the general progress of the profession, and that of the collateral sciences, this society has of itself contributed its full share towards elevating and maintaining progress in medicine.

Medical Education.—The elevation of the standard for the Doctorate is one of those vexed problems which require the harmonious and united efforts of the profession, organized as we may be, to work out to a satisfactory conclusion. The subject of medical education, although dilated upon at every annual meeting of this and other medical associations, has not been exhausted, and must continue to engage the thoughtful attention of the profession, until settled upon some satisfactory basis. The difficulty arises, not so much from diversity of sentiment as from a division of interest. So long as there are young men and women striving to enter the profession, and assume its responsible duties, whether qualified or not, they will possibly find a way; but it will depend upon ourselves whether the ignorant and unworthy among them shall be admitted to our membership. In the language of our code of eithics, "a physician ought to be imbuded with the greatness of his mission, and the responsibilities he habitually incurs in its discharge." Hood has beautifully expressed this sentiment in his str.ctures upon the Hahnemanic Vagaries:

"Above all price of wealth
The Body's jewel—not for minds profane,
Or hands, to tamper with in practice vain—
Like to a woman's virtue is man's health,
A Heavenly gift within a holy shrine!
To be approached and touched with serious fear,
By hands made pure, and hearts of faith severe,
Ev'n as the Priesthood of the One Divine."

Reform in medical education must commence with ourselves as well as the schools. No practitioner who regards his professional character and standing, should ever permit his name to be used as a preceptor unless he devotes sufficient time to the daily examination of his student upon the subject matter of his reading, as will satisfy himself that the student thoroughly understands it. It is only by such a course that the preceptor can ascertain the necessary capacity of his student. While a knowledge of Latin and Greek may be conceded as profitable and beneficial, yet it cannot be regarded as so essential that the student should be a classical scholar, as that he should have the mental ability necessary to grasp and comprehend the difficult problems involved in the science and practice of medicine. If we bring this question home, and understand our own professional reputation is to be reflected to a great extent in our students, we would hesitate in undertaking a task which would be liable to bring discredit upon ourselves. So far as my own observation extends, very few of us have the time to devote to the proper teaching of the student which is necessary to enable him to do us credit; the consequence is, that he plods along as best he can, and the preceptor is hardly aware how ignorant or unqualified the student may be when he enters the profession. Were the practice of receiving students wholly ignored by the active practitioner, and the business left to such experts as could find the time, and had the other qualifications necessary to do the student justice, an enormous stride would be made in establishing the basis of medical education; and the incompetent student would be weeded out before a sufficient growth had been attained by him in medical science, to enable him to enter the schools. If every member of this society were to adhere strictly to these rules, large numbers of those who now apply to us to enter the profession would be rejected at the threshold, and the complaints against the schools for turning out poorly qualified graduates would cease. I am aware that this reform involves a herculean effort on the part of the profession, both here and elsewhere; but I believe that it is an effort which is demanded for the best interest of the public, and will be sustained by the wise and prudent among the profession.

The tendency toward specialism in practice was referred to, to some extent, and the desire to engage in special practice without a general knowledge of medicine condemned.

The desirability of forming auxillary societies, county and district, was urged upon the members of the society.

In relation to sanitary regulations he spoke as follows:

How to lessen sickness and death is a question which may well invite our closest attention. English statisticians estimate that each death in the census has been preceded by six hundred and thirty-six days of sickness during the whole life of the individual. The census statistics show that 500,000 deaths occur annually in the United States, and in our own State they may be computed at about 30,000. Applying the English estimate, we have the enormous number of 19,080,000 days of sickness for the people of the State each year.

The statesman or legislator who views these facts in their relation to the economies of labor, will not fail to see that the industrial population constitute about one-third of the whole number; and that there must be a positive loss of $20,000,000 annually by loss of time, and expenses incurred, in consequence of such sickness, among that class alone. While the arbitrary condition of society makes it certain that the sick and enfeebled must be supported by the healthy.

The lessening of sickness and mortality depends very greatly upon our knowledge of the causes of disease, the investigation of which is one of great labor, and skill, and many of these causes are of so subtle a nature as to elude the vigilance of any single individual, or community; and, if the people of the State (who are to be benefited) would practically reap the advantage to be gained by such knowledge, they must supply the central force, or bureau of health, with which we may co-operate.

It is not arrogance on our part to say that the world is indebted to our profession for the little of sanitary knowledge now known, and which is steadily increasing the average duration of human life, and lessening the number of days of sickness. Unaided by the discoveries in medical science, and their application to the wants of man in this new country, the West would have been this day undeveloped, instead of containing, as it does, a dense and cultured population.

Were our profession to give up the task of sanitary enlightenment, where is the world to get it? Certainly not from the various *isms* and *pathies* in medicine, whose brief lifetime hardly suffices to make a generation, in the ages since medicine has had a written history.

In this age and country, when enjoyment may be said to have a money value, the people are not slow to forward objects which will promote that enjoyment and culture; and they appreciate health, whether considered in its pecuniary or philanthropic aspects, I regard it as one of the best means of discouraging quackery, to teach the people sanitary knowledge, as by this means they will be better able to distinguish the true from the false system of medicine, and *expose* the pretender to knowledge not possessed. There would be some propriety in referring the subject matter of the different *isms* and *pathies* to a committee to be reported on annually, by which means some benefit would accrue to our people in giving them lessons upon this branch of sanitary science, were it not proved during the long history of medicine that each false system soon gives way to another equally absurd.

While our Christian civilization has greatly improved, and benefited the race morally and intellectually, it has utterly failed to eradicate many of the evils of our social system. Living, as we do, in the same atmosphere of the whole people, and observing the evidences which daily present themselves only to the physicians, we can speak with more assurance of the prevalence of those evils which are known only to a few, besides the individual himself, but which afflict a greater number than the public know of, or are willing to admit. Whether scrofula, struma, tuberculosis, and some other forms of disease are the offspring of syphilis is not yet determined, but enough is established to prove the wide-spread and detrimental influence which this disease has upon the race. Every physician knows that a large per centage of the mortality of infantile and adult life, is due to the indirect influences of this destroying agent, which is probably greater than that from any one of the epidemic diseases which have been called "the scourges of mankind," and yet its name scarcely appears in the records of mortuary reports.

I am aware that there is great prejudice on the part of the community (and particularly of the religious portion of it,) to the discussion of this subject, and all attempts on the part of municipalities, in this country, to exercise police supervision against the spread of this disease, has met with opposition from those who assume to be teachers in morals. The very able report upon this subject to the American Medical Association, by Dr. Gross, of Philadelphia, takes strong ground in favor of the enactment of laws looking to its suppression. I think the same views are entertained by most of those who are familiar with the havoc and suffering inflicted upon the human race in consequence of this evil, and it would seem that no false modesty, nor deference to the prejudices of others, should lead us to ignore the subject, or prevent us from freely imparting that information which will contribute to the advantage of society. Our Legislators and Boards of Health provide summary means for the abatement of nuisances detrimental to the life and health of the people. The evil goes beyond the individual or community in which it exists, and contaminates the vital blood for unborn generations. No position is so high as to be exempt from its unseen influences, and no people of the habitable globe but have felt its destroying blight.

Ancient Greece, the home and birth place of Philosophy and Letters, was so fettered by her religious prejudices, that she prevented the study of anatomy, because it desecrated the dead. We should allow no similar reverence for a sentiment to interfere with efforts for saving the world from so destructive an evil.

The want of properly instructed nurses is universally felt, and it is to be hoped that some plan may be divised by which their proper training may become general, and not confined to the larger cities, where hospital facilities exist. We all know how much depends upon the skill and care of those having charge of the sick in the absence of the physician; and that our efforts in behalf of our patients are often neutralized by the want of intelligent nursing. It would seem to me from my observation, that this deficiency is so wide-spread, and the demand so urgent, as to warrant the establishment of training schools in every populous county of the State.

In conclusion, let us not forget that we are celebrating our 30th anniversary upon one of the most beautiful islands of Lake Erie, where but little more than sixty years ago, the great American commander beheld from the placid bay in front of us, the British ships with which he hastened to engage in deadly strife. Our great State was then comparatively a wilderness, with here and there a hardy settlement of pioneers, determined to hew out a civilization for their decendants, in spite of opposing obstacles. Could the immortal Perry now behold the transformation which has been effected in a single lifetime, he would say that those pioneers had not lived in vain. Adopting the talismanic words of the dying Lawrence, he inscribed them upon his banner. So let us, relying in full faith upon earnest endeavor to achieve progress in medicine, cheer each other onward with his motto, "DON'T GIVE UP THE SHIP."

The officers of the two nations who have lost their lives in that battle here quietly sleep together in peace, with their resting place hallowed as the scene of one of the world's great naval contests for the maintenance of a principle which equally benefits the vanquished and the victor. What a more fitting place than this for promoting the homogenity of the medical profession of a continent?

Away from the excitements of the busy city, fanned by the breezes of the great lakes, and surrounded by their health-giving waters, all can forget for a brief period the cares which suffering humanity impose, and gain new strength as we tread the pleasant groves of this favorite watering place, while we draw from that fountain of wisdom and experience, THE PROFFESSION OF THE STATE."

The meeting of the society was an interesting one, and several valuable papers were read which called forth instructive discussions.

The officers elected for the following year are: President, Dr. E. Williams, Cincinnati; Vice-Presidents, Dr. W. S. Scarf, Bellefontaine; Dr. A. H. Agard, Sandusky; Dr. S. S. Thorn, Toledo; Dr. J. L. Beach, West Jefferson; Secretary, Dr. J. W. Haddock, Cincinnati; Treasurer, Dr. S. S. Gray, Piqua.

The Society meets at the same place the third Tuesday in June, 1876.

